# The association between cultural and social occasions and smoking cessation: The case of Saudi Arabia

**DOI:** 10.18332/tid/174490

**Published:** 2023-11-17

**Authors:** Sarah S. Monshi, Mohammed M. Alqahtani, Abdulaziz S. Alangari, Tassnym H. Sinky, Abdulmohsen H. Al-Zalabani, Abdullah M. Alanazi

**Affiliations:** 1 Department of Health Services Management, College of Public Health and Health Informatics, Umm Al-Qura University, Makkah, Saudi Arabia; 2 Department of Respiratory Therapy, College of Applied Medical Sciences, King Saud bin Abdulaziz University for Health Sciences, Riyadh, Saudi Arabia; 3 King Abdullah International Medical Research Center, Riyadh, Saudi Arabia; 4 Department of Epidemiology and Biostatistics, College of Public Health and Health Informatics, King Saud bin Abdulaziz University for Health Sciences, Riyadh, Saudi Arabia; 5 Department of Health Promotion and Education, College of Public Health and Health Informatics, Umm Al-Qura University, Makkah, Saudi Arabia; 6 Department of Family and Community Medicine, College of Medicine, Taibah University, Medina, Saudi Arabia

**Keywords:** culture, occasion, smoking cessation, intention to quit

## Abstract

**INTRODUCTION:**

Tobacco use remains high in Saudi Arabia, necessitating an understanding of the influence of cultural and social events on smoking cessation. This study examined whether cultural and social events like Ramadan, Eids, and birthdays, motivate Saudi adults to quit smoking.

**METHODS:**

Convenience sampling of 742 Saudi tobacco users was conducted between July 2021 and May 2022. A cross-sectional survey was used to assess self-reported desire to quit (1 = ‘least desire’ to 10 = ‘highest desire’) and behaviors in relation to special occasions. Multiple linear regression was performed to examine the association between cultural and social occasions and the desire to quit, while logistic regression was used to assess the relationship between cultural and social occasions and historical quit attempts, medicinal nicotine product use, visiting smoking cessation clinics, and calling the national Quitline. Demographic variables and tobacco types were controlled for in the analyses.

**RESULTS:**

Considering quitting during Ramadan, Eids, and birthdays was associated with a higher desire to quit (Ramadan: B=2.99; 95% CI: 2.51–3.47, Eids: B=2.83; 95% CI: 2.12–3.54, and birthdays: B=2.76; 95% CI: 1.93–3.60, p<0.01) and a greater likelihood of past quit attempts (Ramadan: AOR=8.2; 95% CI: 5.5–12.1, Eids: AOR=5.8; 95% CI: 3.2–10.5, and birthdays: AOR=4.8; 95% CI: 2.4–9.4). Only considering quitting during Ramadan was associated with calling the national Quitline (AOR=2.9; 95% CI: 1.8–4.9).

**CONCLUSIONS:**

Cultural and social events like Ramadan and Eids motivate adults in Saudi Arabia to attempt tobacco cessation. Targeting interventions around meaningful cultural occasions may promote successful quitting. These findings highlight the importance of understanding cultural and religious influences on cessation behaviors in Saudi Arabia and in other countries.

## INTRODUCTION

Tobacco use remains the leading preventable cause of death worldwide, causing more than 8 million deaths annually worldwide^1^. Tobacco use, therefore, has been considered a health-related risk. The Surgeon General’s Report (2014) revealed that smoking has long been associated with adverse effects on the respiratory and cardiovascular system, causing chronic lung and heart diseases^2^. Moreover, there is ample evidence that tobacco use may increase the risk of cancer^2^. In addition, tobacco use has adverse effects on the health outcomes of the musculoskeletal system^3^. It has also been displayed that tobacco use may yield a lower health-related quality of life^4^.

It has been reported that 19.8% of the Saudi population are currently tobacco users^5^. The percentage of current tobacco use is higher among males (30%) compared to females (4.2%)^5^. Female smoking is associated with cultural stigma in most Arab countries, including Saudi Arabia. However, the change in women’s role in society, along with the tobacco marketing activities, have contributed to normalizing smoking among females in Saudi Arabia^6^. Since 2010, 20.9% of girls aged 13–15 years and living in Saudi Arabia, who had never tried tobacco products, showed a willingness to initiate tobacco use in the future^7^.

The most common tobacco products in Saudi Arabia are cigarettes and waterpipe^5^. Emerging tobacco products such as electronic nicotine delivery systems (ENDS) have also been reported^5^. The economic losses from tobacco use in Saudi Arabia accounted for US$ 17.2 billion^8^, and there are approximately 70000 individuals who die annually due to tobacco-related morbidity^9^. Due to increased tobacco use and health hazards, there is a great need for more effective and tailored smoking cessation interventions. Evidence indicates that if smokers stopped smoking, they could lessen their risk of tobacco-related morbidity and mortality, and potentially gain up to ten years of life^10^.

Success in quitting smoking depends on several factors. Intention to quit is one factor responsible for 12% of the variance in successful quit rate^11^. Other factors that contribute to successful quitting are nicotine dependence, history of quit attempts, health consciousness, cultural norms, social support, clinician assistance, belief in smoking cessation’s benefit, availability of cessation treatment, and implementation of tobacco control measures including increased tobacco prices and prevention of secondhand smoking^12,13^. Due to social stigma related to smoking among women in several Arab countries, factors that influence cessation among women were investigated. Concern about weight gain, self-efficacy, perceived outcome expectations were found to be common predictors for cessation among women^14^.

Efforts to stop smoking and successful quitting among tobacco users are also predicted by the motivation to quit, which could be influenced by cultural factors^15^. Studies show that religious reasons have positively spurred university students to stop smoking^16^. It has been proposed that a religion inspired approach could be used to curtail tobacco use and enhance smoking cessation^17^. This approach can be used with smokers in religion-based societies, such as Islam societies, where individuals believe in the importance of preserving health^18^. There is also a substantial body of scientific evidence that there is an urgent necessity for the role of Islamic beliefs and teachings in influencing individual motivation to abstain from smoking^18^.

Additionally, it is argued that religious events, such as Ramadan and Eids, are times when motivation to refrain from smoking is most potent^18^. Also, it has been demonstrated that engaging in social occasions may help smokers to abstain from smoking^19^. However, little is known concerning the relationship between social and cultural occasions such as Ramadan and Eids and the intention to quit smoking among adults in Saudi Arabia.

This study assessed the relationship between cultural occasions, such as the month of Ramadan and the days of Eids, and the intention to quit smoking in the Saudi population. We hypothesized that there would be a positive association between social and cultural occasions and the intention to quit smoking among adults in Saudi Arabia. Our findings may contribute to the development of effective policies and programs to instigate smoking cessation interventions.

## METHODS

### Study design and sample

Data were collected from July 2021 through May 2022 using a cross-sectional design. In all, 742 Saudis aged ≥ 18 years and who were currently using tobacco products, including [cigarettes, waterpipes, or ENDS such as electronic cigarettes (e-cigarettes)] were recruited using a convenience sampling technique via word of mouth and an anonymous online survey. Unfortunately, this approach made it challenging to determine the study’s response rate. Participants were considered current users of tobacco if they had used cigarettes, waterpipe, or ENDS at least once in the last 30 days. The survey consisted of an informed consent request and closed-ended questions. Because of the social context related to the study questions, the authors consulted experts in the field to validate each question. The survey was pretested and piloted among a small group of the target population (n=20 adult Saudi tobacco users) to assess the clarity of the questions. To attain a 5% margin of error and a 95% confidence level within a reference population exceeding 20000 adults, it was established that a minimum sample size of 377 tobacco users was needed. The study was ethically approved by the Institutional Review Board of the King Abdullah International Medical Research Center (SP21R/234/05).

### Measures

*Demographic characteristics*

Demographic characteristics were collected from participants, including age, sex (female, male), education level (high school or lower, diploma degree, Bachelor’s degree or higher), and employment status (unemployed, student, employed).

*Cultural and social occasions*

Ramadan was considered a cultural occasion while Eids and birthdays were considered social occasions. We asked participants several questions that assessed smoking change during cultural and social events, including Ramadan (‘Have you considered quitting tobacco cigarettes/waterpipe/e-cigarettes in Ramadan?’), Eids holidays (‘Have you considered quitting tobacco cigarettes/waterpipe/e-cigarettes in Eids?’), and birthdays (‘Have you considered quitting tobacco cigarettes/waterpipe/e-cigarettes on your birthday?’). The responses to each of the previous questions were dichotomized into (0=No, 1=Yes).

*Smoking behavior characteristics*

To assess smoking behavior characteristics, we used various author-constructed questions that included the desire to quit with response options: ‘What is your desire to quit?’ with responses from 1 = ‘least desire’ to 10 = ‘highest desire’); historical quit attempts, ‘Have you ever tried to quit tobacco cigarettes/waterpipe/e-cigarettes in the past?’ with response yes/no); medicinal nicotine product use, ‘Have you used medicinal nicotine products such as nicotine patches and nicotine gum?’ with response yes/no); visiting smoking cessation clinic, ‘Have you visited a smoking cessation clinic?’ with response: yes/no; and calling the national Quitline, ‘Have you called smoking cessation clinics?’ with response yes/no. In addition, participants were asked about their type of substance use (cigarettes, waterpipe, and e-cigarettes) and the frequency of use, with response yes/no. These measures were used as a covariate in the regression analyses.

### Data analysis

Descriptive statistics for categorical variables (frequency and percentages) and continuous variables (means and standard deviation) were reported to represent participant characteristics. Multiple linear regression models were used to assess the association between cultural and social occasions and the desire to quit. In addition, multiple binomial logistic regression models were also used to examine the association between cultural and social occasions and historical quit attempts, medicinal nicotine product use, visiting smoking cessation clinics, and calling the national Quitline. The models were controlled for demographic characteristics including age, gender, education level, employment status, and marital status) and type of substance use. Potential predictors were selected based on literature for their association. Statistical significance was set at p<0.05 using two-sided tests. All analyses were performed using SAS^®^ software version 9.4 (SAS Institute Inc., Cary, NC, USA).

## RESULTS

The average age of the sample was 25.1 years (SD=7.0), and the majority of the respondents were male (92.4%) and single (74.4%) (Supplementary file). The most commonly reported type of tobacco used was cigarettes (45.4%), followed by waterpipe (27.4%), and e-cigarettes (27.2%). The sample comprised 44.4% with a Bachelor’s degree or higher, 39.5% with a high school degree or lower, and 16.1% with a diploma. Most of the participants were employed (44.1%) or students (38.6%), and only 17.3% were unemployed.

The average score for desire to quit was 5.8 ± 3.4 (Table 1). In addition, the majority of the participants indicated historical quit attempts (56.7%). However, the majority of the sample did not use medicinal nicotine products (85.5%), did not visit a smoking cessation clinic (86.1%), or called the national Quitline (87.5%). Quitting because of cultural and social events was found to have differing results. Considering quitting smoking on birthdays and Eids was only found to be (10.2%) and (14.7%), respectively. Considering quitting during the Ramadan month, however, had a higher prevalence (46.9%).

**Table 1:** Quitting characteristics of tobacco users, Saudi Arabia, July 2021–May 2022 (N=742)

*Characteristics*	*n*	*%*
**Desire to quit** (score) (N=685), mean ± SD	5.8 ± 3.4	
**Lifetime quit** (N=716)		
No	310	43.3
Yes	406	56.7
**Medicinal nicotine products use** (N=705)		
No	603	85.5
Yes	102	14.5
**Visited smoking cessation clinic** (N=705)		
No	607	86.1
Yes	98	13.9
**Quitting smoking in Ramadan** (N=715)		
No	380	53.1
Yes	335	46.9
**Quitting smoking in Eids** (N=713)		
No	608	85.3
Yes	105	14.7
**Quitting smoking on birthday** (N=708)		
No	636	89.8
Yes	72	10.2
**Called the national Quitline** (N=703)		
No	615	87.5
Yes	88	12.5

After controlling for demographic factors in the multiple linear regression models, those who considered quitting during Ramadan (B=2.99; 95% CI: 2.51–3.47, p<0.01), Eids (B=2.83; 95% CI: 2.12–3.54, p<0.01), and birthdays (B=2.76; 95% CI: 1.93–3.60, p<0.01) had a higher desire to quit scores compared to those who did not consider quitting during these occasions (Table 2). Tobacco type was significantly associated with the desire to quit score. On average, waterpipe and e-cigarettes users had less desire to quit than those who smoked cigarettes. Regarding demographic variables, only age and education level were significantly associated with the desire to quit. As age increased, the average desire to quit score increased, and those with a Diploma had a higher desire to quit than those with Bachelor’s and higher degree.

**Table 2:** The association between characteristics of tobacco users, quitting occasions, and desire to quit, Saudi Arabia, July 2021–May 2022 (N=742)

*Variables*	*Desire to quit tobacco*											
	*Model 1*				*Model 2*				*Model 3*			
	*B*	*95% CI*	*β*	*p*	*B*	*95% CI*	*β*	*p*	*B*	*95% CI*	*β*	*p*
**Age (years)**	0.05	0.00–0.11	0.11	0.06	0.07	0.02–0.13	0.15	0.01*	0.08	0.02–0.14	0.15	0.02*
**Gender**												
Female vs male	-0.85	-1.77–0.06	-0.06	0.07	-0.82	-1.78–0.14	-0.06	0.09	-0.41	-1.37–0.56	-0.03	0.41
**Education level**												
High school and lower vs Bachelor’s and higher	0.56	-0.01–1.12	0.08	0.05	0.51	-0.01–1.10	0.07	0.10	0.68	0.07–1.29	0.10	0.03*
Diploma vs Bachelor’s and higher	1.19	0.49–1.89	0.13	<0.01*	0.83	0.08–1.58	0.09	0.03*	1.00	0.24–1.76	0.11	0.01*
**Employment status**												
Student vs employed	0.24	-0.41–0.89	0.03	0.35	0.22	-0.48–0.92	0.03	0.54	0.15	-0.56–0.86	0.02	0.67
Unemployed vs employed	-0.35	-1.07–0.38	-0.04	0.47	-0.46	-1.22–0.31	-0.05	0.25	-0.55	-1.33–0.24	-0.06	0.17
**Marital status**												
Married vs single	-0.37	-1.20–0.46	-0.05	0.38	-0.46	-1.35–0.42	-0.06	0.30	-0.51	-1.43–0.40	-0.07	0.27
Divorced vs single	-1.00	-3.07–1.07	-0.03	0.34	0.31	-1.71–2.33	0.01	0.76	-0.60	-2.76–1.55	-0.02	0.58
**Tobacco type**												
Shisha vs cigarette	-1.06	-1.68 – -0.44	-0.14	<0.01*	-2.06	-2.72 – -1.41	-0.27	<0.01*	-1.89	-2.56 – -1.23	-0.25	<0.01*
Vaping vs cigarette	-0.56	-1.15–0.03	-0.07	0.06	-1.35	-1.96 – -0.73	-0.18	<0.01*	-1.18	-1.81 – -0.56	-0.16	<0.01*
**Quitting in Ramadan**												
Yes vs No	2.99	2.51–3.47	0.43	<0.01*								
**Quitting in Eids**												
Yes vs No					2.83	2.12–3.54	0.29	<0.01*				
**Quitting on birthday**												
Yes vs No									2.76	1.93–3.60	0.24	<0.01*
R^2^	0.08				0.15				0.12			

Multiple linear regression analyses include age, gender, education level, occupation, marital status, tobacco type, quitting in Ramadan (Model 1 only), quitting in Eids (Model 2 only), quitting on your birthday (Model 3 only).

* Statistical significance at p<0.05.

Similarly, those who considered quitting smoking in social and cultural events were significantly more likely to have historical quit attempts (Ramadan: AOR=8.2; 95% CI: 5.5–12.1, Eids: AOR=5.8; 95% CI: 3.2–10.5, and birthdays: AOR=4.8; 95% CI: 2.4–9.4), visit smoking cessation clinics (Ramadan: AOR=2.0; 95% CI: 1.2–3.2, Eids: AOR=2.4; 95% CI: 1.4–4.4, and birthdays: AOR=2.1; 95% CI: 1.1–4.0), and use medicinal nicotine products (Ramadan: AOR=3.2; 95% CI: 1.9–5.2, Eids: AOR=3.2; 95% CI: 1.8–5.6, and birthdays: AOR=2.4; 95% CI: 1.3–4.5) than those who did not consider quitting during social and cultural occasions (Table 3). Only those who considered quitting in Ramadan were significantly associated with calling the national Quitline (AOR=2.9; 95% CI: 1.8–4.9).

**Table 3:** The association between quitting occasions and tobacco users’ quitting behavior, Saudi Arabia, July 2021–May 2022 (N=742)

*Variable*	*Lifetime quit*			*Visited the smoking cessation clinic*			*Medicinal nicotine products use*			*Called the national Quitline*		
	*AOR*	*95% CI*	*p*	*AOR*	*95% CI*	*p*	*AOR*	*95% CI*	*p*	*AOR*	*95% CI*	*p*
**Quitting in Ramadan**			<0.01*			<0.01*			<0.01*			<0.01*
No (Ref.)	1			1			1			1		
Yes	8.2	5.5–12.1		2.0	1.2–3.2		3.2	1.9–5.2		2.9	1.8–4.9	
**Quitting in Eids**			<0.01*			<0.01*			<0.01*			0.08
No (Ref.)	1			1			1			1		
Yes	5.8	3.2–10.5		2.4	1.4–4.4		3.2	1.8–5.6		1.7	0.9–3.2	
**Quitting on birthday**			<0.01*			0.03*			<0.01*			0.1
No (Ref.)	1			1			1			1		
Yes	4.8	2.4–9.4		2.1	1.1–4.0		2.4	1.3–4.5		1.7	0.8–3.4	

AOR: adjusted odds ratio; adjusted for age, gender, education level, occupation, marital status, and tobacco type (marital status was excluded from ‘visited smoking cessation clinic’ model due to the small sample size in the subgroup).

* Statistical significance at p<0.05, two-sided test.

## DISCUSSION

To our knowledge, this study is the first to examine the role of cultural and social occasions in the desire to quit smoking among tobacco users in Saudi Arabia. Most tobacco users, mainly cigarette users, had a history of quitting smoking yet lacked the use of smoking cessation supports such as the national Quitline, cessation medications, and behavioral therapy. A statistical relationship was found between social and cultural occasions (such as birthdays, Ramadan, and Eids) and the desire and planning to quit smoking. Planning to quit smoking at social and cultural occasions increased the likelihood of utilizing smoking cessation services and medications. Of note, the likelihood of calling the national Quitline increased among smokers who only planned to quit during Ramadan.

This study found that the likelihood of desire to quit smoking increased among cigarette users compared to waterpipe and e-cigarettes users. A similar pattern was reported in previous studies^20^. Waterpipe and e-cigarettes are perceived as alternative safer tobacco products and are sometimes used to help smokers quit combustible cigarette smoking^21,22^. This could explain the lack of interest in quitting waterpipes and e-cigarettes in this study. In addition, in the Arab world, waterpipe use is strongly related to the social context^6^. It is perceived to create an environment of cohesiveness where people can socially engage in an inexpensive activity^23^. Therefore, quitting waterpipe in a collectivist society like Saudi society may reduce social utility^24^. Thus, it is recommended that smoking cessation interventions are designed based on sociocultural values, as are embedded in Saudi Arabia. For example, instead of focusing only on educating people about the risks of smoking on individuals’ health, interventions may also focus on the negative impact of smoking based on social values^24^.

Our results show that more than half of the participants who tried quitting smoking did not seek cessation support, including Quitline consultation, cessation medications, or behavioral therapy. Several reasons may lead smokers to go without any quit assistance, such as misinformation about smoking cessation pharmacotherapy^25^, inaccessible cessation services, lack of support from healthcare providers^12^, or concern related to the privacy of disclosing tobacco use and/or using cessation aids^26^. Previous research suggested that unsuccessful quit experiences led to a lack of cessation aid utilization^27^. On the other hand, our findings suggest that social and cultural events increase the likelihood of quit attempts and seeking assistance to reach complete abstinence by visiting smoking cessation clinics and using medicinal nicotine products. Cultural occasions, such as Ramadan, may play a role in the decision-making process among smokers to enhance religious commitment that calls for preserving health^18^. However, previous studies indicate that religious commitment during a specific time may fade after the event ends^28^. This contradiction between studies’ results requires more attention from policymakers and researchers to explore reasons for unassisted quitting in Saudi Arabia, examine the social and cultural context behind smoking cessation interventions, and investigate the role of religion in different communities to maintain smoking abstinence successfully.

It is not uncommon for smokers to plan to quit smoking on particular seasons and social occasions such as birthdays, anniversaries, Christmas, and the New Year^29,30^. This phenomenon has occurred due to the intense anti-tobacco campaigns that center on social events such as the New Year (i.e. ‘New Year quit smoking resolution’) to encourage smokers to quit smoking^29^. Our study also found higher quit attempts during cultural occasions such as Ramadan and Eids among smokers in Saudi Arabia and a significant relationship between the desire to quit smoking and planning to quit smoking during these events. Similar to the findings in Saudi Arabia, reduced tobacco consumption and increased quit attempts during Ramadan were observed in Muslims in Malaysia, the United Kingdom, Qatar, and the United States^31-33^. Compared to Eids and birthdays, the prevalence of quitting during Ramadan was higher (46.9%). This finding could be explained by the occasions’ duration: thirty days for Ramadan, three days or less for Eids , and birthdays usually one day. Consequently, the odds of quit attempts may be higher during cultural and social occasions that last for a longer period. This possibility should be investigated to enhance tailoring smoking cessation interventions in Saudi Arabia.

Several reasons could explain the increase in quit attempts during Ramadan and Eids observed in this study. Since tobacco use is considered forbidden in Islam^34^, smokers may use Ramadan to quit smoking as a resolution to avoid disliked behaviors. Secondly, quitting smoking is accompanied by withdrawal symptoms^35^. Smokers, therefore, may take advantage of the fast during Ramadan to overcome the withdrawal symptoms associated with quitting. Finally, multiple tobacco control activities have been designed to target smoking during Ramadan^31-33,36^. This may increase awareness against tobacco use and promote the use of smoking cessation supports such as smoking cessation consultation services (i.e. Quitline calls and clinic visits) and smoking cessation medications. Despite the promising outcomes related to smoking cessation during religious events, the previous review indicates insufficient evidence supporting religion-based interventions to quit smoking, arguing that religious commitments may dissolve by the end of the social or cultural occasion, leading to smoking relapse^18^.

Moreover, in this study, the desire to quit smoking during the Eids occasions may be explained by the impact of Ramadan cessation resolutions. In contrast to our findings, smoking relapse was previously reported during Eids because the celebrations triggered smokers to smoke^32^. Although Eids are special events, no study has examined their impact on smoking patterns and quitting. This is an area of improvement for tobacco control researchers interested in the effect of sociocultural occasions on tobacco use and the design of culturally tailored interventions.

### Limitations

This study has several limitations. First, the cross-sectional study design prevents us from understanding the temporal sequence of cultural events and smoking cessation intentions and behaviors. Second, the sample was limited to tobacco users in Saudi Arabia. The recruitment methods did not collect information about history of tobacco use (ever vs current tobacco use) and did not yield a random sample, so the findings may not be generalizable to other populations, countries, or cultures. The recruitment strategy along with the sampling technique in this study hinder our ability to determine the response rate. Also, in the sample, only 7.6% were women; thus, findings may be inadequate to interpret the relationship between cultural events and smoking cessation behavior among women in Saudi Arabia. Third, self-reported measures of tobacco use and quitting intentions may be subject to recall and social desirability biases. Finally, this study was only able to examine intentions to quit and failed quit attempts in relation to cultural and social occasions. However, this study lacked data about lifetime tobacco use (current vs ever smoking) and on whether participants successfully quit tobacco use during these events. Future research should prospectively follow participants to determine actual quit rates related to cultural and social events.

## CONCLUSIONS

This study provides initial insights into the relationship between social and cultural events and tobacco cessation in Saudi Arabia. Findings suggest that such occasions motivate tobacco users to plan and attempt to quit tobacco. In particular, quitting during Ramadan was associated with higher use of cessation services such as calling the national Quitline. Despite the limitations, this study highlights the importance of cultural sensitivity in tobacco control. Interventions that tap into meaningful cultural and social occasions may be particularly impactful in Saudi Arabia and in other countries.

## Data Availability

The data supporting this research are available from the authors on reasonable request.
