# Supplementary material for: The association between cultural and social occasions and smoking cessation: The case of Saudi Arabia
Source: Tob Induc Dis. 2023 Nov 17;21:153. doi: 10.18332/tid/174490 (PMC10655210; doi:10.18332/tid/174490)
Supplement: Supplementary file 1 [file TID-21-153-s1.pdf]

Supplementary Material:

Appendix 1. Demographic characteristics of sample participants, Saudi Arabia, [July 2021-May 2022] (n=742)

Variable	Mean	±SD
**Age (years) (n=705)**	25.1	7.0
**Variable**	**n**	**%**
**Gender (n=732)**		
Female	56	7.6
Male	676	92.4
**Educational attainment (n=734)**		
High school and lower	290	39.5
Diploma	118	16.1
Bachelor and above	326	44.4
**Occupation (n=734)**		
Have a job	324	44.1
Do not have a job	127	17.3
Student	283	38.6
**Marital status (n=733)**		
Single	545	74.4
Married	175	23.9
Divorced	13	1.8
Widowed	0	0.0
**Tobacco type (n=742)**		
Cigarette	337	45.4
Waterpipe	203	27.4
E-cigarettes	202	27.2

©2023 Monshi S.S. et al.
